# AIM™ platform: A new immunotherapy approach for viral diseases

**DOI:** 10.3389/fmed.2022.1070529

**Published:** 2022-12-23

**Authors:** David Langan, Ruipeng Wang, Keshanti Tidwell, Selome Mitiku, Alison Farrell, Catrina Johnson, Adam Parks, Lauren Suarez, Shweta Jain, Sojung Kim, Kristi Jones, Mathias Oelke, Jerome Zeldis

**Affiliations:** NexImmune Inc., Gaithersburg, MD, United States

**Keywords:** T cell, ACT, AIM, aAPC, NexImmune, viral, immunotherapy, nanoparticle

## Abstract

In addition to complications of acute diseases, chronic viral infections are linked to both malignancies and autoimmune disorders. Lack of adequate treatment options for Epstein-Barr virus (EBV), Human T-lymphotropic virus type 1 (HTLV-1), and human papillomavirus (HPV) remains. The NexImmune Artificial Immune Modulation (AIM) nanoparticle platform can be used to direct T cell responses by mimicking the dendritic cell function. In one application, AIM nanoparticles are used ex vivo to enrich and expand (E+E) rare populations of multi-antigen-specific CD8^+^ T cells for use of these cells as an AIM adoptive cell therapy. This study has demonstrated using E+E CD8^+^ T cells, the functional relevance of targeting EBV, HTLV-1, and HPV. Expanded T cells consist primarily of effector memory, central memory, and self-renewing stem-like memory T cells directed at selected viral antigen peptides presented by the AIM nanoparticle. T cells expanded against either EBV- or HPV-antigens were highly polyfunctional and displayed substantial in vitro cytotoxic activity against cell lines expressing the respective antigens. Our initial work was in the context of exploring T cells expanded from healthy donors and restricted to human leukocyte antigen (HLA)-A*02:01 serotype. AIM Adoptive Cell Therapies (ACT) are also being developed for other HLA class I serotypes. AIM adoptive cell therapies of autologous or allogeneic T cells specific to antigens associated with acute myeloid leukemia and multiple myeloma are currently in the clinic. The utility and flexibility of the AIM nanoparticle platform will be expanded as we advance the second application, an AIM injectable off-the-shelf nanoparticle, which targets multiple antigen-specific T cell populations to either activate, tolerize, or destroy these targeted CD8^+^ T cells directly in vivo, leaving non-target cells alone. The AIM injectable platform offers the potential to develop new multi-antigen specific therapies for treating infectious diseases, cancer, and autoimmune diseases.

## Introduction

Many viral infections, if not cleared during the acute stages, may become chronic. Chronic infections are often associated with intermittent recrudescence, autoimmune complications and/or malignancies (1–5). Both the innate and acquired immune systems are involved. Antigen-presenting cells (APCs), especially dendritic cells (DCs), can modulate both types of immunity by displaying antigen derived peptides and cytokine release to direct antigen-specific T cell and other immune cells. In this report, we will describe our work utilizing AIM nanoparticles as artificial APCs (aAPCs), or synthetic DCs, designed to present antigenic peptides in the context of class I human leukocyte antigen (HLA) and simultaneously deliver a second co-stimulation signal to expand antigen-specific T cells for adoptive cell therapies (ACT). In a second application, the AIM nanoparticle is the therapeutic. Injectable nanoparticles with signal 1 (peptide loaded class I HLA) and signal 2 (co-stimulatory anti-CD28) proteins activate and expand viral antigen-specific CD8^+^ T cells without affecting non-antigen-specific cells. Both the aAPC and AIM injectable approaches simultaneously engage antigen-specific T cells through their peptide loaded HLA molecule and a co-stimulatory second signal that can up or down regulate the function of the engaged T cell. As described below, these approaches offer potential treatments of a broad range of viral diseases.

Adoptive cell therapies has shown increasing promise for treating Epstein-Barr virus (EBV) and Cytomegalovirus (CMV) infections, specifically in immune compromised patients after allogeneic stem cell transplantation (2, 6, 7). These patients may benefit from virus-specific ACT used either prophylactically or therapeutically (2). Additionally, both preclinical models and clinical trials using ACT have supported its utility to treat virally driven malignancies associated with human papillomavirus (HPV) and EBV (2–4). EBV targeted ACT might further elucidate links between EBV infection and multiple sclerosis (5). Multiple challenges need to be addressed for ACTs to expand on early successes. These challenges include addressing disease escape mechanisms, increasing T cell persistence, antigenic heterogeneity including emergence of neo-antigens, reducing on-target off-tissue adverse events, reducing manufacturing cost, and product inconsistency (8, 9).

**FIGURE 1:**
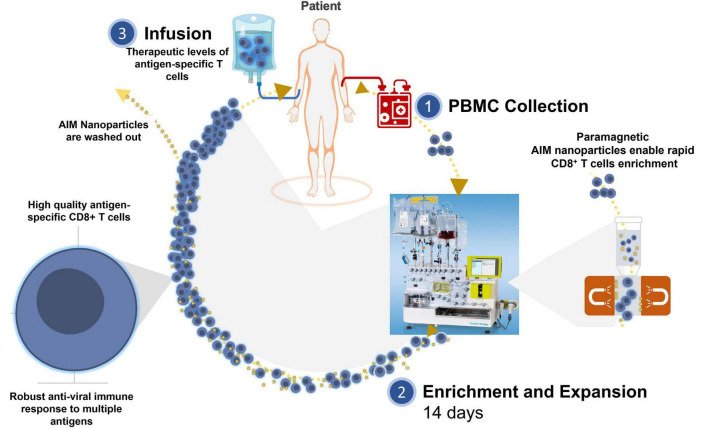
Artificial Immune Modulation Adoptive Cell Therapy (AIM ACT). (1) Leukapheresis material is collected from a patient with infection (*e.g.*, EBV, HTLV-1, HPV, and HIV). (2) The CD8^+^ T cells are enriched for using paramagnetic AIM nanoparticles that function as artificial APCs (aAPCs) to expand CD8^+^ T cells specific for immunodominant viral antigens. (3) After 14 days expansion, AIM nanoparticles are washed out and the AIM ACT is ready to be infused into patients.

With the Artificial Immune Modulation (AIM) ACT system, NexImmune may overcome many of these challenges to develop cellular therapies for patients with virally driven diseases (Figure 1). Incorporation of magnetic based systems to generate infectious disease specific ACT have shown promise (6, 7), for reasons including the reduction in the manufacturing time compared to other clinically tested methods (10, 11). The AIM Enrichment and Expansion (E+E) system is a reproducible, closed manufacturing process that consistently produces T cells for ACT comprised of non-genetically engineered multiple antigen specific effector and long-lived memory T cells from autologous or allogeneic donor leukopaks (12). Two AIM ACT clinical trials are currently ongoing: one for relapsed/refractory multiple myeloma (MM) [NCT04505813] and another for acute myeloid leukemia (AML) [NCT04284228]. An additional trial for HPV-related Head and Neck cancer will be initiated.

Here we provide an overview of the AIM E+E system used to manufacture T cells for ACT, and report preclinical results from using this system to expand CD8^+^ T cells directed against viral antigens of Epstein-Barr virus (EBV), Human T-lymphotropic virus type 1 (HTLV-1), or high-risk human papillomavirus (HPV) types 16 and 18 from HLA-A*02:01 healthy donor cells. Each of the final AIM ACT products consists of a mixture of CD3^+^/CD4^–^ T cells that are mostly specific for multiple antigens from EBV, HTLV-1 or HPV with greater than 90% of these T cells being of the T cell memory phenotype [*i.e.*, effector memory (Tem), central memory (Tcm), or stem cell-like memory (Tscm)]. In addition, EBV-specific AIM ACT cells demonstrate functional activity in terms of antigen-specific cytotoxic killing and cytokine profile.

## Materials and methods

### Nanoparticles, reagents, and cell lines

Briefly, nanoparticles were manufactured by direct conjugation of humanized HLA-Ig dimer and anti-CD28 antibody to MACS Microbeads (Miltenyi Biotec, Bergisch Gladbach, Germany) as described previously (13). Antibodies used for flow cytometry were purchased from Miltenyi Biotec and are described in Supplementary Table 1. Cell lines were originally purchased from American Type Culture Collection (ATCC, Manassas, VA). To generate red fluorescence protein (mKate2) expressing cell lines for Incucyte studies, cells were transduced with Incucyte NucLight Red Lentivirus (EF1a, Puromycin) and selected under 2 μg/ml puromycin (Sartorius, Goettingen, Germany). The amino acid sequence of HLA-A*02:01 and beta-2-Microglobulin (ß2M) (GenBank AYV97550.1 and AAA51811.1) were codon optimized and cloned downstream of separate EF1α promoters in a PiggyBac cDNA cloning vector (PB533A-2, System Biosciences, Palo Alto, CA). The expression vector was co-transfected into HeLa cells along with a PiggyBac transposase helper plasmid from System Biosciences (PB210PA-1) to mediate transposition into the host genome for stable cellular expression of membrane-bound HLA-A*02:01 and ß2M. Cells were passaged in 1 mg/ml G418 for selection of stable expression from the cell lines (Supplementary Figure 1). CellEvent Caspase-3/7 Green Detection Reagent, CellTrace Far Red, and Sytox Blue Dead Cell Stain were purchased from Thermo Fisher Scientific.

### Enrichment and expansion of T cells

Leukapheresis mononuclear cells from HLA-A*02:01 healthy donors were obtained from commercial vendors. Donors were not tested for EBV, HTLV-1, or HPV. Cells were processed according to the NexImmune AIM platform to produce an ACT, as previously described (14). As previously described, on day 0, after CD4^+^ T cell depletion, CD8^+^ T cells were enriched on a CliniMacs Prodigy (Miltenyi Biotec) using AIM nanoparticles loaded with the peptides listed in Table 1 (12). The enriched cells were seeded into a G-Rex culture system (Wilson-Wolf Manufacturing) and expanded for 14 days. The E+E cells were collected on Day 14 to freeze and store in liquid nitrogen until further testing.

**TABLE 1:** HLA-A2 restricted peptides from viral driven diseases tested in the AIM ACT system.

Virus	Name	Protein	Sequence	Diseases
HTLV-1	Tax LLF	Tax	LLFGYPVYV (20)	Adult T-cell leukemia/Lymphoma (ATL), HTLV-1-associated myelopathy
HTLV-1	Tax SFH	Tax	SFHSLHLLF (19)	
HTLV-1	GPP ALL	Pol	ALLGEIQWV (55)	
HTLV-1	GPP SLI	Pol	SLISHGLPV (55)	
HTLV-1	GPP FMQ	Gag	FMQTIRLAV (55)	
EBV	LMP2 CLG	LMP2	CLGGLLTMV (56)	Infectious mononucleosis, Multiple sclerosis, chronic active EBV (CAEBV), lymphoproliferative disease, certain malignancies including lymphomas and carcinomas
EBV	LMP2 FLY	LMP2	FLYALALLL (57)	
EBV	BMLF1	BMLF1	GLCTLVAML (58)	
EBV	BRLF1	BRLF1	YVLDHLIVV (59)	
EBV	EBNA3	EBNA3	LLDFVRFMGV (60)	
EBV	LMP1	LMP1	YLQQNWWTL (61)	
HPV-16	HPV2	E7	YMLDLQPETT (62)	Squamous cell carcinomas (*e.g.*, head and neck, cervical, anal, penile), warts
HPV-16	HPV21	E6	TIHDIILEC (63)	
HPV-18	HPV38	E6	LFVVYRDSI	
HPV-18	HPV42	E7	FQQLFLNTL (64)	
N/A	SVN1	Human /BIRC5	QMFFCFKEL (65)	

### Cell counting and flow cytometry analysis

Donor cells were counted with the NucleoCounter NC-200 (Chemometec, Lillerod, Denmark). Dead cells were stained with Zombie Aqua for 10 min in the dark. Surface staining of cells was done at 4°C for 10 min with anti-human antibodies in PBS supplemented with 2% FBS and 0.01% sodium azide (FACS Buffer). Cells were then washed once with FACS buffer before analyzing. For the intracellular cytokine assay, cells were stained with anti-CD107a overnight during the period of activation. Then cells were washed once with PBS before staining with Zombie Aqua (BioLegend, San Diego, CA) and washed again with FACS buffer before surface staining as described above. Next cells were fixed and permeabilized with 1X eBiosciences Fixation/Permeabilization solution. Intracellular staining was performed with antibodies in 1X eBioscience Permeabilization Buffer. Cells were then washed twice with FACS Buffer and analyzed. For the flow-based killing assay, nuclear RFP expressed endogenously was used to detect tumor cells and CellTrace Far Red was used to detect PBMCs. Cells were analyzed on a MacsQuant10 or MacsQuant16 flow cytometer (Miltenyi Biotec). Data was analyzed with FlowLogic software (Inivai Technologies, Mentone, Australia).

### Culturing tumor cell lines and E+E cells

Cell lines were cultured in RPMI supplemented with 10% fetal bovine serum (16140-071), 1 mM sodium pyruvate (11360070), 1X MEM non-essential amino acids (11140-035), 1X 2-Mercapthoethanol (21985-023), and 1X MEM Vitamin (11120052). These Gibco cell culture reagents were purchased from Thermo Fisher Scientific (Waltham, MA). Human PBMCs or E+E cells were thawed from cryopreservation the day before *in vitro* assay testing and allowed to rest overnight in TexMACS medium (Miltenyi Biotec) with cytokine supplements (14). The following day, tumor cells, PBMCs, or E+E cells were prepared in single suspension with fresh media before performing specific assays as described below.

### Intracellular cytokine staining (ICS) assay

E+E cells were prepared in fresh TexMACS the day after thawing and 1.0x10^5^ cells/well were seeded into a 96-well round-bottom plate (Corning, Corning, NY). Either peptide loaded nanoparticles or phorbol 12-myristate 13-acetate/Ionomycin in Invitrogen’s Cell Stimulation Cocktail (plus protein transport inhibitors, 500X) (Thermo Fisher Scientific) were used to stimulate cells. Cells alone with Invitrogen’s eBioscience Protein Transport Inhibitor Cocktail (Thermo Fisher Scientific) were used as the unstimulated control and when stimulating cells with peptide loaded nanoparticles. Cells were fixed, permeabilized, and stained before analyzing as described above.

### *In vitro* incucyte-based killing assay

Incucyte compatible tumor cell lines expressing red fluorescence protein (mKate2) were generated by transducing cells with Incucyte NucLight Red Lentivirus (EF1a, Puromycin) according to manufactures recommendation (Sartorius). Tumor cells were seeded at 5.0*10^3^ cells per well in a clear 96-well flat-bottom plates (Corning) the evening prior to starting a killing assay. The following day E+E cells were added to wells to achieve the desired effector to target ratio. For assessment of antigen-specific killing by E+E cells, A375 tumor cells were pulsed with an EBV peptide or irrelevant peptide (10 μg/ml) 2-4 h prior to adding the E+E cells. For negative controls no E+E cells were added. Phase-contrast and fluorescent images were taken every 4 h using the Incucyte S3 (Sartorius). IncuCyte S3 2019A software was used to analyze images. From each image RFP- puncta corresponding to tumor cell nuclei were enumerated and reported as relative cell number normalized to the starting cell number.

### Flow cytometry caspase-3/7-based killing assay

Autologous donor PBMCs were washed with non-supplemented RPMI and stained at 37°C for 20 min with CellTrace Far Red (0.1 μM). After staining, cells were washed twice with supplemented RPMI. Tumor cells expressing nuclear RFP were dissociated into single cell suspension using Accutase solution (STEMCELL Technologies) and washed with supplemented RPMI. Donor PBMCs or tumor cells were then added to 96-well round-bottom plates (Corning) at 5.0*10^4^. E+E cells were then added to wells to achieve the indicated E:T ratio. Controls received no E+E cells. CellEvent Caspase-3/7 Green was added to each well (250 nM final). After culturing at 37°C for 4-7 h, SYTOX Blue dead cell stain was added (0.5 μM final) and plates were incubated in the dark for 5 min before flow cytometry analysis.

### Calculations and statistical analysis

The percentage of antigen-specific killing in the incucyte killing assays was calculated as follows:


$$\%\,A\,n\,t\,i\,g\,e\,n\,\text{-}\,s\,p\,e\,c\,i\,f\,i\,c\,k\,i\,l\,l\,i\,n\,g=\frac{\begin{array}{c} (Mean\%tumorcellnumberincontrols\\ -\%tumorcellnumberwithpeptidepulsed) \end{array}}{\left(M\,e\,a\,n\%\,t\,u\,m\,o\,r\,c\,e\,l\,l\,n\,u\,m\,b\,e\,r\,i\,n\,c\,o\,n\,t\,r\,o\,l\,s\right)}$$

The percentage of caspase 3/7 positive tumor or autologous PBMC cells was calculated as follows:


$$\%\,C\,a\,s\,p\,a\,s\,e\,3/7\,p\,o\,s\,i\,t\,i\,v\,e=\frac{\begin{array}{c} (\%positivewithE+Ecells\\ -Mean\%positiveincontrols) \end{array}}{\left(100\%-M\,e\,a\,n\%\,p\,o\,s\,i\,t\,i\,v\,e\,i\,n\,c\,o\,n\,t\,r\,o\,l\,s\right)}$$

Statistical difference between groups was tested by either student T-test or ratio paired T test as indicated using GraphPad Prism 9 software. Significant difference is reported where p-values are ≤ 0.5.

## Results

### HTLV-1 and EBV antigen specificity of AIM E+E T cells

The list of HLA-A2 restricted EBV, HTLV-1, and HPV peptides used in the AIM E+E manufacturing system are listed in Table 1.

**FIGURE 2:**
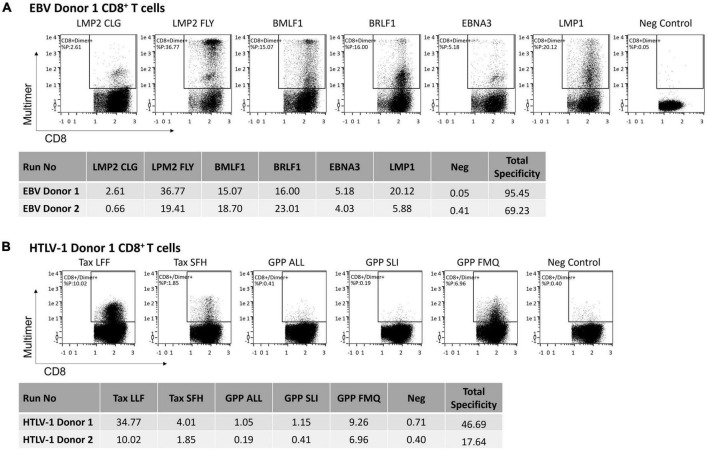
The frequency of antigen-specific CD8^+^ T cells among cells enriched and expanded to target HLA-A2 restricted viral peptides. **(A)** EBV peptides (6 total) or **(B)** HTLV-1 peptides (5 total) were used to generate E+E cells from two healthy donors. Reported is the frequency of CD8^+^ T cells that bind peptide-loaded HLA-A2-Ig dimer. Also shown is the total frequency of CD8^+^ T cells to each of the EBV or HTLV-1 peptides. Total frequency is normalized using an irrelevant peptide, the negative control.

Using the AIM E+E system with EBV peptides, the final expanded T cells included significant numbers of antigen-specific CD8^+^ T cells to both lytic (BMLF1 and BRLF1) and latent (LMP1, LMP2, and EBNA3) EBV antigens. Six EBV peptides from the following proteins LMP2, BMLF1, BRLF1, EBNA3, and LMP1 were used to generate AIM ACT from two healthy donors. The estimated frequency of the E+E CD8^+^ T cells to each EBV peptide ranged from 0.25% to 36.77% (Figure 2A). The total frequency of all cells to the six EBV peptides was 95.45% for EBV Donor 1 and 69.23% for EBV Donor 2. Among the E+E cells from both donors, the frequencies to LMP2 FLY (36.77% and 19.0%), BMLF1 (18.29% and 15.02%), and BRLF1 (22.6% and 15.95%) were the highest, whereas the frequency to LMP2 CLG (2.56% and 0.25%) was relatively low.

Using the AIM E+E system with HTLV-1 peptides, the summed total frequency of E+E CD8^+^ T cells to all five HTLV-1 peptides was 46.69% for Donor 1 and 17.64% for Donor 2. The frequency to each antigen ranged from 0 to 34.06% (Figure 2B). The frequencies to Tax LLF (33.06% and 9.62%), GPP FMQ (8.55 and 6.56%), and Tax SFH (3.3% and 1.45%) were the highest among expanded cells from both donors, whereas CD8^+^ T cells showed a relatively low frequency to GPP ALL (0.34% and 0.0%) and GPP SLI (0.44 and 0.01%).

### Memory phenotype of AIM E+E T cells

For a T cell ACT to be potent and durable, it will ideally consist of a heterogeneous population of memory T cell phenotypes that can combine cytotoxic capacity with long-term persistence and immunologic memory (12). We define Memory T cells as those with the phenotype of either Tem (CD62L^–^CD45RA^–^), Tcm (CD62L^+^CD45RA^–^), or Tscm (CD62L^+^CD45RA^+^CD95^+^).

**FIGURE 3:**
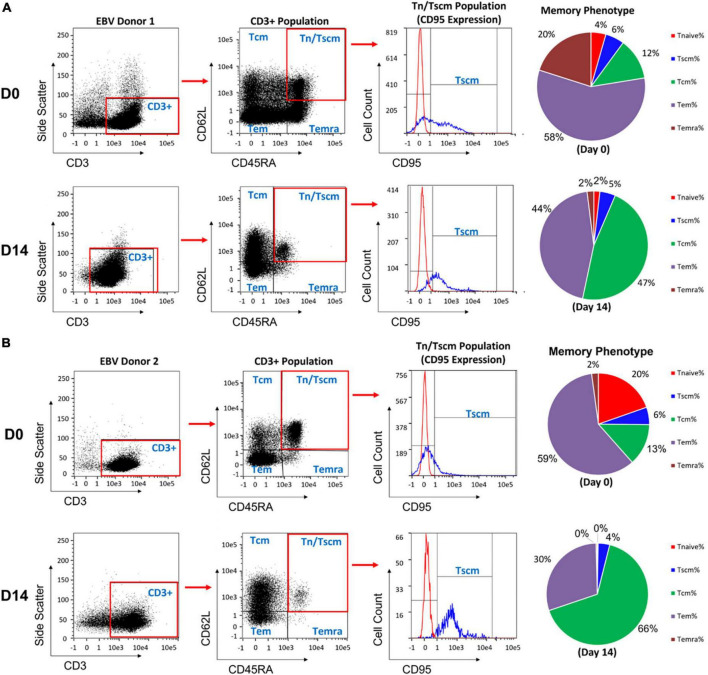
Phenotype of the T cells on day 0, before expansion, and on day 14 after enrichment and expansion for EBV antigen specific T cells using the AIM ACT system. **(A,B)** Results are shown from two healthy donors. After completion of expansion, T cells from each run were taken for phenotyping memory cell populations by staining for CD62L vs CD45RA. The naïve and stem cell-like memory cells were further distinguished by CD95 expression. The pie charts show the distribution of memory T cell subsets at day 0 and day 14 of the process. Central memory T cells (Tcm), effector memory T cells (Tem), effector memory T cells re-expressing CD45RA (Temra), stem cell-like memory T cells (Tscm), naïve T cell (Tn).

The AIM platform consistently expanded clinically relevant numbers of T cells containing greater than 90% Tscm, Tcm, and Tem cells in 14-days. Figure 3 displays the memory phenotypes of T cells post-enrichment from leukopaks from two healthy donors and again on day 14 after expansion using EBV peptide loaded nanoparticles. The Tem compartment was the largest subset (58-59%) among the T cells immediately post-enrichment. After expansion (Day 14), the Tem subset of total cells was reduced to 30-44% with a shift in phenotype favoring the Tcm subtype from 12-13% to 47-66% of total cells. Tcm cells are the primary reservoir of proliferating T cells and are responsible for immunologic memory. Of the expanded total T cells, expression of CD95 by some CD62L^+^CD45RA^+^ T cells showed that 4-5% were Tscm. Both naïve T cells (Tn) and terminally differentiated effector memory T cells expressing CD45RA (Temra) cells constitute less than 5% of the total T cell population.

### Polyfunctional activity of EBV specific AIM E+E T cells

**FIGURE 4:**
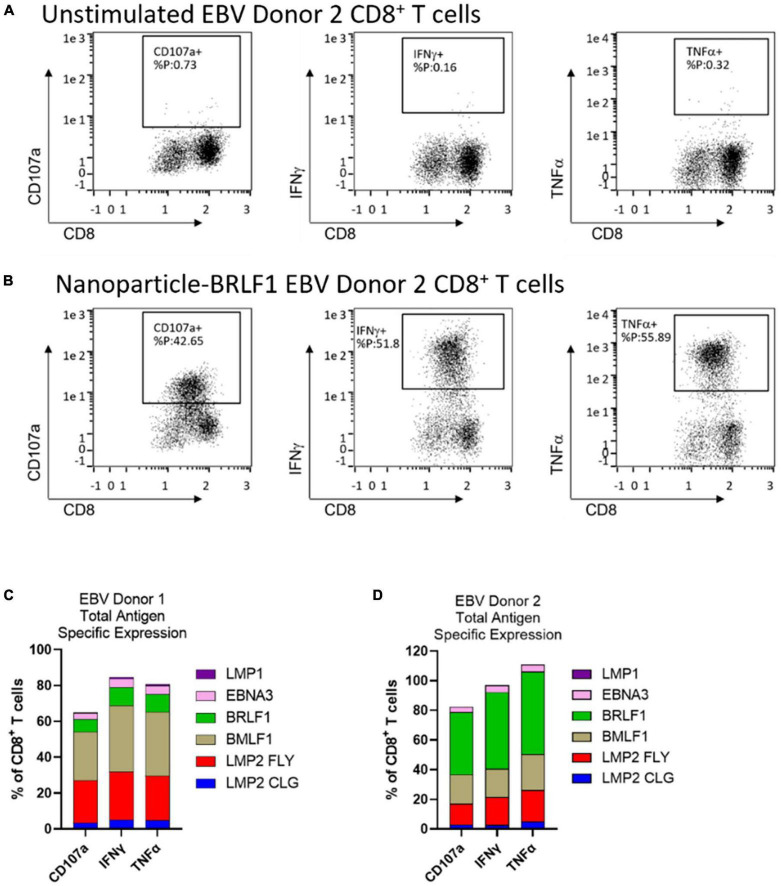
AIM ACT cells selected on 6 EBV peptides produce a polyfunctional response upon antigen-specific restimulation. Intracellular cytokine staining was run on EBV antigen-specific E+E cells from 2 healthy donors. Cells were left unstimulated or were restimulated with nanoparticles loaded with one of the six EBV peptides. Representative plots show the expression of functional markers by CD8^+^ T cells from EBV Donor 2 when **(A)** left unstimulated or when **(B)** stimulated with BRLF1 loaded nanoparticles. The cumulative percentage of CD8^+^ T cells from **(C)** EBV Donor 1 and **(D)** EBV Donor 2 that express each functional marker in response to stimulation with peptide loaded nanoparticles. Conditions were run in duplicate wells.

EBV-specific AIM ACT cells from two healthy donors were generated using AIM nanoparticles loaded with the 6 different EBV antigen-peptides described below and in Table 1. Assessment by intracellular cytokine staining (ICS) showed that most of the T cells responded to a peptide by expressing Type1 cytokines, IFNγ and TNFα, and by displaying surface CD107a (Figure 4). Although there was T cell specificity to LMP1 (5.47 and 20.07%), upon stimulation with LMP1 loaded nanoparticles very few cells expressed cytokines. The total percentage of CD8^+^ T cells that responded to these EBV peptides ranged from 80 to 100%.

### Antigen specific cytotoxicity of AIM E+E cells

**FIGURE 5:**
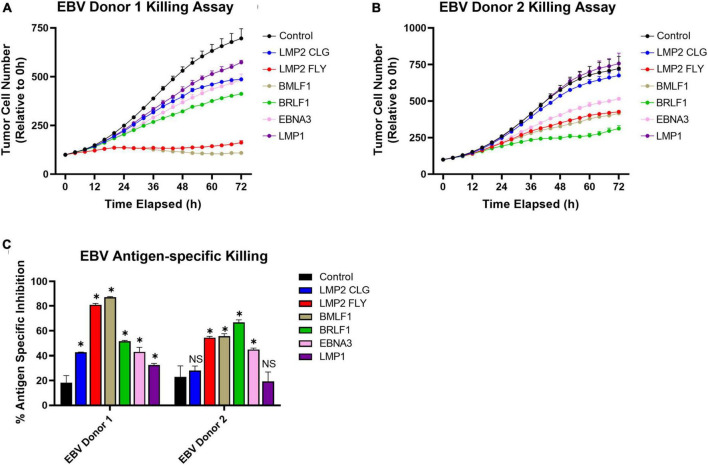
Multi-antigen specific killing by EBV specific E+E cells from 2 healthy donors. A375 target cells were pulsed with peptides (10 ug/ml); one of six peptides used to enrich and expand T cells, a control HLA-A2-restricted peptide, or no peptide. Then T cells were added to coculture for 72 h at an effector to target ratio of 10:1. Target cells were enumerated by fluorescent microscopy to identify RFP expressing live A375 cells. **(A,B)** Reported is the relative number of tumor cells compared just after adding E+E cells (0 h). **(C)** Reported is the percentage reduction in tumor cell number at 72 h compared to when no effector cells were added. Statistical difference was determined by student T test comparing the relative tumor cell number at 72 h for each condition with when the control peptide was pulsed (*p* > 0.05 = NS, *p ≤ 0.05). Conditions were run in duplicate wells.

Adoptive cell therapies that targets multiple viral antigens may help to overcome issues that arise from circulating T cell heterogeneity between donors and antigenic heterogeneity of infected cells. This heterogeneous immune response is illustrated by our observations made from leukopaks from two health donors. EBV-specific expanded T cells were cocultured with HLA-A2 positive target cells that were pulsed with one of the six EBV peptides used for enrichment and expansion. The expanded T cells from Donor 1 had antigen-specific cytotoxic activity directed to all six peptides but the cells from Donor 2 were directed to only 4 peptides. The greatest cytotoxic activity was directed against LMP2 FLY, BLMF1, and BRLF1 (Figure 5). This corresponds to the same peptides for which the highest frequencies (Figure 2) and polyfunctional activities (Figure 4) were observed.

**FIGURE 6:**
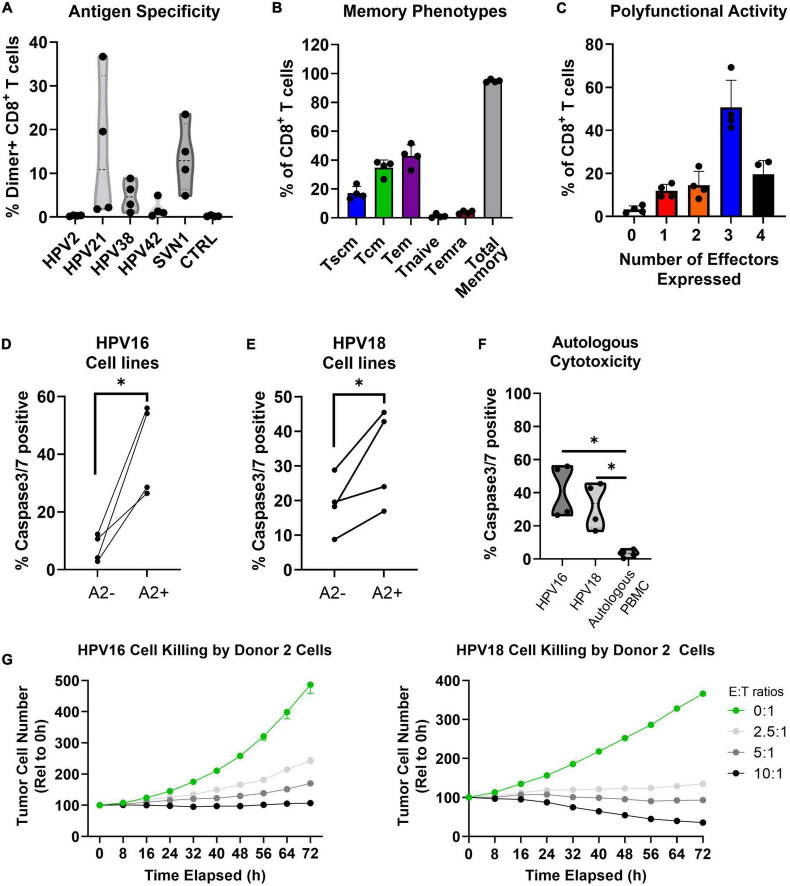
In the preclinical validation of NEXI003, the AIM platform consistently generated high quality cytotoxic T cells for HPV-associated cancer treatment. Results are reported from four independent runs for the validation of NEXI-003 (*n* = 4). **(A)** The percentage of peptide specific (Dimer+) cells among CD8^+^ T cells (Mean ± StdDev). **(B)** The memory phenotype of CD8^+^ T cells. **(C)** The percentage of CD8^+^ T cells that express 0-4 total functional markers (*i.e*., CD107a, IL-2, IFNγ, and TNFα) upon stimulation with PMA/Ionomycin (Mean ± StdDev). **(D–F)** The percentage of live target cells that were caspase-3/7 positive after coculture with E+E cells at a 10:1 E:T ratio. Target cells that were HLA-A2 positive (*i.e.*, CaSki or HeLa-A2) are directly compared to those that were HLA-A2 negative (*i.e*., SiHa or HeLa) or were autologous PBMCs. Statistical difference tested by ratio paired T test (*p ≤ 0.05). **(G)** HPV-16^+^ or HPV-18^+^ HLA-A2 expressing cell lines (*i.e.*, CaSki or HeLa-A2, respectively) were cultured for 72 h alone or with E+E cells from Donor 2 at E:T ratios of 2.5:1-10:1. Reported is the tumor cell number relative to the 0 h (Mean ± SEM; *n* = 3). Effector cell to target cell ratio (E:T ratio).

NEXI-003 is an ACT therapy targeting HPV-related cancers. The HPV-16 and HPV-18 strains are targeted using peptides from the immunodominant E6 and E7 antigens of these strains and by adding a fifth peptide from the tumor-associated antigen Survivin. In Table 2 and Figure 6, the numerical, phenotypic, and functional characterization of the preclinical runs (*n* = 4) for NEXI-003 are summarized. In Supplementary Figures 2, 3, the gating strategy used to determine the frequencies of these cell subsets are shown with representative FACS plots. These expanded cells consisted of highly pure populations of CD3^+^/CD4^–^ T cells, greater than 90% (96.82% ± 2.89%). Cells were 69% ± 13% CD8^+^ T cells and these CD8^+^ T cells showed multi-antigen specificity to these HPV-related cancer antigens with a total frequency ranging from 17.66% to 56.34%.

**TABLE 2:** With the AIM platform a highly pure population of T cells is consistently generated for NEXI-003.

Donor	Viability	CD3^+^ %	CD8^+^ T cell %	γδ T cell %	CD4^–^ T cell %
Donor 1	94.6	96.63	83.86	5.43	95.36
Donor 2	88.6	96.15	60.13	32.89	95.53
Donor 3	88.1	98.48	76.69	15.04	96.29
Donor 4	89.7	99.48	56.34	39.92	99.33

* CD3^+^ %, CD8^+^ T cell %, and CD4^–^ T cell % from identity staining panel.

* γδ T cells % taken from TCR staining panel.

### Preclinical validation of NEXI-003: An AIM ACT to treat HPV-related cancer

The mean total memory phenotype of the T cells was 95% (Figure 6B) which is consistent with what has been observed with the EBV-antigen expanded AIM ACT (Figure 3), as well as other AIM ACT against HTLV-1 and various tumor-associated antigens (Data not shown). An average of 71% of T cells produce 3-4 functional markers (*i.e.*, CD107a, IL-2, IFNγ, and TNFα) upon non-specific stimulation with PMA/ionomycin (Figure 6C). The AIM ACT cells consistently showed cytotoxic activity directed at both HPV-16 and HPV-18 positive HLA-A2 expressing tumor cells lines (Figures 6D, E). *In vitro* testing of the HPV-related cancer specific T cells determined that no significant cytotoxic activity was directed at autologous PBMCs (Figure 6F). In Figure 6G, a representative killing assay with HPV Donor 2 expanded cells shows sustained cytotoxic activity *in vitro* over 72 h.

## Discussion

Central to the AIM ACT application and used to manufacture ACT products for virally driven diseases are paramagnetic nanoparticles that are designed to display HLA-Ig dimer molecules loaded with disease relevant peptides (signal 1) as well as co-stimulatory anti-CD28 antibody (signal 2). Antigen peptide loaded AIM nanoparticles selectively enrich, activate, and expand populations of antigen-specific CD8^+^ T cells from the naïve and memory repertoire (15). AIM nanoparticles were previously shown to elicit T cell responses to a dominant peptide from CMV, pp65 (NLVPMVATV) (16). Ongoing experiments to evaluate AIM nanoparticles loaded with different viral peptides are focused on EBV, HTLV-1, and HPV. The intention is to expand this work to other viruses including HIV.

Most adults have been infected with EBV at some time during their life. Often adolescent and adult infections result in mononucleosis. CD8^+^ T cell responses to both lytic and latent antigens occur, but at a higher frequency for some lytic antigens (17, 18). Our results are consistent with previous findings that show the peptides we tested are immunodominant antigens in donors that are likely to have been previously infected with EBV. Multiple experiments are ongoing to characterize and compare the final T cells derived from EBV infected patients with or without EBV-related disorders. HTLV-1 viral infections are associated with adult T cell leukemia/lymphoma (ATL) and a progressive neurological disorder known as HTLV-1 associated myelopathy or tropical spastic paraparesis (HAM/TSP). Developing an AIM ACT against the HTLV-1 virally infected cells is showing promise. The antigen peptide targets we used have been described and show that peptides from the Tax 1 protein are immunodominant and predominant to those of Gag and polymerase (Pol) (19, 20).

The AIM E+E clinical system employed in these experiments is a two-step closed process that uses the CliniMacs Prodigy (Miltenyi) for the enrichment of antigen-specific CD8^+^ T cells and the GREX culture system for expansion of the specific CD8^+^ T cells over a 14 day period (Figure 1). Since the AIM nanoparticles present only HLA class I restricted peptides and CD4^+^ T cells have been found to expand non-specifically under these culture conditions eventually outgrowing the antigen-specific CD8^+^ T cells of interest, CD4^+^ T cells are initially removed. This also removes potential regulatory T cells from the culture and therefore from the final product, which could impact the expansion of the antigen-specific T cells as well as the functionality of the ACT. In murine models, regulatory T cells have been shown to restrict CD8^+^ T cell memory and inhibit tumorigenic immunity elicited by CD8^+^ and CD3^+^/CD4^–^CD8^–^ T cells (21, 22). AIM ACT consist of ≥ 90% CD3^+^/CD8^+^ and CD3^+^/CD4^–^CD8^–^ T cells, the latter being primarily gd T cells. Several recent reviews highlight the increasing interest in using gd T cells for cancer treatment and more recently for viral infections (23–26). In our AIM ACT trials we have confirmed that E+E cells are well tolerated with no induced graft-vs-host disease (GVHD) or other grade 3-4 adverse events while observing clinical activity and persistence of our infused antigen-specific CD8^+^ T cells (27). Different methods of generating ACT for viral diseases have been applied in the clinic with some clinical success (2–4, 6, 7). In a small clinical trial with 10 HSCT patients, monocyte-derived DC expanded allogeneic donor-derived T cells specific to 4 viruses were given a median of 63 days post-transplant. No reactivation of EBV, adenovirus, or varicella zoster virus was observed and 6 of 10 patients showed CMV reactivation (28). Only 1 patient required additional antiviral therapy after T cell infusion and no patient died during the 12 months of follow-up (28). Patients displayed anti-viral immunity against all 4 viruses after infusion. Also, grade II-IV acute GVHD occurred in 3 of the patients following T cell infusion. This supports the use of ACT for such disease treatment and prevention.

While some *ex vivo* T cell expansion protocols produce large numbers of T cells, in many cases these cells include more Temra cells with short-lived clinical potency. It is believed that terminally differentiated Temra are short-lived and die soon after transfer and antigen exposure. Temra cells are a significant proportion of the DC mediated T cell expanded products and may not be able to control malignancies on a long-term basis due to lack of persistence (29–31). This was also shown with an ACT for viral infections. CMV-seropositive HSCT patients infused with less differentiated CMV-specific memory T cells were conferred prolonged protection from CMV-reactivation compared to patients that received more terminally differentiated memory cells (32). The less differentiated T cell compartments (*i.e.*, Tscm and Tcm) are capable of self-renewal and replenish the Tem compartment cells. This is critical for immunological memory and the persistence of ACT T cells after infusion (32, 33). These less differentiated subsets of T cells associated with long-term survival are among the T cell populations that are enriched and expanded using the AIM platform. In murine and non-human primate models, CD8^+^ T cells with a less differentiated memory phenotype (similar to human Tscm) engraft more efficiently and persist longer *in vivo* (33–36). Ichikawa et al. compared cell expansion systems using anti-CD3/CD28 Dynabeads, autologous DCs, or AIM nanoparticles to expand MART-1 antigen-specific CD8^+^ T cells from melanoma patients (14). MART1-specific CD8^+^ T cells demonstrated higher expansion using DCs and AIM nanoparticles vs. Dynabeads, approximately 1,000-fold from pre-enriched numbers. Expansion conditions with Dynabeads may not have been optimal but may also perform better when antigen-specific expansion is unnecessary such as with genetically modified cell therapies, like CAR T cell systems (37). The percentage of MART-1 specific expanded cells on day 14 was 10-times greater with AIM nanoparticles than either DCs or Dynabeads. Not only did the AIM nanoparticle provide the highest antigen-specific cell numbers, but these cells also had a greater percentage of self-renewing Tscm, longer telomers, and less terminally differentiated T cells than DC expanded cells from the same donors. Noteworthy, the resulting phenotype is the same whether the starting population comes from a healthy donor or a donor with cancer (14). Similarly, the EBV-antigen specific cells that are enriched at D0 are likely to have a predominance in memory phenotypes, as compared to antigen-specific T cells for other AIM ACT indications, because greater than 90% of the general population has been exposed to EBV. These results suggest that previous exposure to a virus may not detract from the ability to generate AIM ACT with memory T cells, including Tscm that are functional and capable of eliminating infected cells.

In addition to therapeutic safety and persistence, a potent anti-viral response is important to clinical success. The anti-viral or anti-cancer activity of CD8^+^ T cells is mediated by a polyfunctional Type1 cytokine response (*e.g.*, IL-2, IFNγ, and TNFα) (38, 39). Autologous IFNγ signaling, for example, increases CD8^+^ T cell numbers and cytotoxic activity (40, 41). In a separate study comparing aAPCs and monocyte-derived DC expanded viral antigen-specific T cells, aAPC expanded T cells had a greater percentage of polyfunctional cells expressing Type1 cytokines (42). The T cell polyfunctional response appeared to be regulated, in part, by the initial method of expansion. Monocyte-derived DC expanded T cells showed signs of senescence or exhaustion after multiple rounds of stimulation whereas cells initially activated with aAPC sustained their polyfunctional activity. To control latent virus reemergence, a sustained polyfunctional anti-viral activity is important for preventing disease recrudescence. The total frequency of CD8^+^ T cells with a Type1 cytokine response to these EBV peptides was equivalent to or greater than the total frequency of EBV peptide-specific cells estimated by multimer staining. The observation from testing EBV Donor 2 E+E cells suggests that some antigen-specific cells may not be detected by peptide loaded multimer staining and that the frequency of cells to some of these peptides may be higher than reported in Figure 2. LMP1 specific T cells response to LMP1 loaded nanoparticles was low. Recognition of peptide: HLA by CD8^+^ T cells can occur without inducing a strong functional response and activation during expansion can lead to exhaustion. The identification of clinically relevant CD8^+^ T cells targeting LMP1 as well as ACT specific to LMP1 has proven difficult (17, 18). LMP1 remains a promising clinical target because of its expression alongside LMP2, for which we observed strong functional responses from T cells, in tumors without EBV lytic proteins expressed, such as type II latency tumors. In patients with EBV-associated tumors that received an ACT of LMP1/2-specific CD8+ T cell, 28 of 29 patients receiving this ACT as an adjuvant therapy remained in remission at a median of 3.1 years after CTL infusion and of 21 patients with relapsed or resistant disease at the time of CTL infusion, 11 had complete responses (43). Further characterization of the T cell responses to LMP1 and LMP2 by ELISPOT analysis showed that many patients had fewer T cells respond to LMP1 than LMP2 and that the response to LMP1 often waned relatively quickly after infusion.

Two AIM ACT clinical trials are ongoing, and the clinical trial for HPV-related Head and Neck cancer will initiate by year’s end. Chronic infection from oncogenic viruses is estimated to contribute to 10% of cancers globally (44, 45). Of the oncogenic viruses, HPV accounts for 4.6-5.2% of cancer, with HPV-16 and HPV-18 being the most prevalent strains associated with cancers of the cervical, vulva, vagina, penis, anus, and oropharynx. Although vaccines are highly effective at preventing disease, HPV-related cancers have low cure rates and relapsed patients have a poor prognosis (44, 46). The E6 and E7 oncogenic proteins of HPV are ubiquitously expressed in tumor cells and as such are ideal targets for ACT. In current therapeutic vaccination and immunotherapeutic strategies E6 and E7 are the primary target antigens (47, 48). A study looking at predictive markers of cervical cancer concluded Survivin was strongly correlated with HPV cervical cancer (49). The overall cytotoxic activity of E+E cells directed at HPV-positive HLA-A2 expressing cell lines did not appear to be correlated to the γδ T cell percentages. Unlike for AIM ACT, γδ T cell expansion protocols often incorporate phosphoantigens recognized by the gammadelta T cell receptor (TCR) and IL-15 from the common gamma-chain receptor family (50, 51). Most of the CD3^+^/CD4^–^CD8^–^ T cells were γδ T cells which have been shown by others to be clinically safe (23, 24), though potential contribution to efficacy is unknown. In clinical trials with AIM ACT for oncological diseases the γδ T cell percentages can vary, and no adverse impact of γδ cells were observed. Also, corroborating the safety of the γδ T cell in ACT are the results showing that high γδ T cell percentages did not contribute to significant autologous PBMC killing (Figure 6F).

The AIM E+E manufacturing system incorporates off-the-shelf nanoparticle technology capable of producing clinically relevant numbers of EBV, HTLV-1, and HPV multi-antigen-specific CD8^+^ T cells from HLA-A*02:01 healthy donor leukopaks. The consistent quantity and quality of final T cells produced are sufficient to support dose escalation Phase I trials for safety and efficacy (cell number data not shown). Multi-antigen-specific CD8^+^ T cells may be of benefit for treating viral infections and their complications including autoimmune diseases and cancers. Work to enrich and expand viral specific CD8^+^ T cells from infected patients is proceeding.

Exploiting the AIM platform E+E system also enables high-throughput screening and selection of relevant peptides for other viral diseases. Four HPV peptides for NEXI-003 were selected from the initial list of 44 HLA-A2 restricted peptides in under 2 months (52). Each peptide was tested using the AIM E+E screening system and evaluated based on the ability to enrich and expand HPV-specific CD8^+^ T cells that can recognize and kill HPV-antigen expressing target cells. These findings for EBV, HTLV-1, and HPV and the previous findings described highlight the potential for using the AIM platform to develop immunotherapies for other viral infections. The flexibility offered by the AIM platform enables substitution of peptides with other viral protein targets and neo-antigens. As a function of the platforms flexibility there is the ability for an individualized immunotherapy that is adapted to a patient’s specific antigen heterogeneity, including a shift in antigen expression over time because of immune pressure.

The AIM platform allows for two types of therapies using AIM nanoparticles: adoptive cell therapy (ACT) and direct injectable therapy (INJ) (Supplementary Figure 4). Due to their long-lived and self-renewing phenotype, once infused into patients these cells (*i.e.*, NEXI-001 and NEXI-002) were found to expand, persist, and traffic to the site of disease. T cell receptor-sequencing (TCR-Seq) with a lower limit of detection of ∼1 in 1 × 10^5^ cells was performed on the T cell products and patient samples before and after lymphodepletion and ACT. The results confirmed that the T cell product contained many dominant T cell clones that were not detected in the patients’ blood at baseline but rapidly expended and persisted after ACT. In addition, multimer staining of the T cell product and patient blood samples after ACT confirmed the presence and expansion of the multimer positive T cells in vivo. While the AIM ACT system expands T cells for infusion *ex vivo*, the AIM INJ system is being developed for direct injection into the patient. Vaccination with peptide pulsed DCs can reconstitute a patient’s anti-viral immunity and is a promising method to treat viral infections (53, 54). The AIM INJ is an off-the-shelf therapy that is designed to directly engage the T cells while bypassing host DC’s that may be compromised in the face of disease. The INJ nanoparticle delivers simultaneous signaling that enables precise engagement, activation, and expansion of effector CD8^+^ T cells to kill infected cells and eliminate acute disease, while providing a memory response for sustained clinical protection. We are currently creating new AIM nanoparticles that incorporate other HLA class I subtypes including *A01, *A03, *A11, *A24, and *B7, allowing for the targeting of a broader patient population. In summary, AIM technology represents a new scalable and cost-effective therapeutic platform that has the potential to cure viral diseases.

## Data availability statement

The raw data supporting the conclusions of this article will be made available by the authors, without undue reservation.

## Author contributions

DL wrote the first draft of the manuscript. RW, LS, SJ, SK, KJ, MO, and JZ contributed to the project conception. DL, RW, KT, SM, AF, and CJ performed the data acquisition. All authors contributed to data analysis, manuscript revision, read, and approved the submitted version.
