# Supplementary material for: AIM™ platform: A new immunotherapy approach for viral diseases
Source: Front Med (Lausanne). 2022 Dec 23;9:1070529. doi: 10.3389/fmed.2022.1070529 (PMC9822776; doi:10.3389/fmed.2022.1070529)
Supplement: Supplementary file 1 [file Data_Sheet_1.pdf]

Supplementary Table.

Supplementary Table 1. List of antibodies used for flow cytometry.

Manufacturer	Reagent	Catalog No.
Miltenyi Biotec	APC anti-human CD3 clone REA613	130-113-135
Miltenyi Biotec	PE anti-human CD4 clone REA623	130-113-225
Miltenyi Biotec	VioBlue anti-human CD8 clone BW135/80	130-113-162
Miltenyi Biotec	PerCP-Vio770 anti-human CD3 clone REA613	130-113-141
Miltenyi Biotec	PE anti-human CD8 clone BW135/80	130-113-158
Miltenyi Biotec	VioBlue anti-human TCR αβ clone REA652	130-119-618
Miltenyi Biotec	FITC anti-human TCR Vδ1 clone REA173	130-118-362
Miltenyi Biotec	APC-Vio770 TCR anti-human Vδ2 clone REA771	130-111-013
Miltenyi Biotec	VioBlue anti-human CD3 clone REA613	130-114-519
Miltenyi Biotec	PerCP-Vio770 anti-human CD45RA clone REA562	130-113-368
Miltenyi Biotec	FITC anti-human CD62L clone 145/15	130-113-619
Miltenyi Biotec	PE anti-human CD95 clone REA738	130-113-004
Miltenyi Biotec	PE anti-human CD3 clone REA613	130-113-139
Miltenyi Biotec	APC Streptavidin	130-106-791
Miltenyi Biotec	FITC anti-human CD107a clone REA792	130-111-620
Miltenyi Biotec	PE anti-human IL-2 clone REA689	130-111-303
Miltenyi Biotec	PE-Vio770 anti-human IFNγ clone REA600	130-113-499
Miltenyi Biotec	APC-Vio770 anti-human TNFα clone aA2	130-120-491

Supplementary Figures

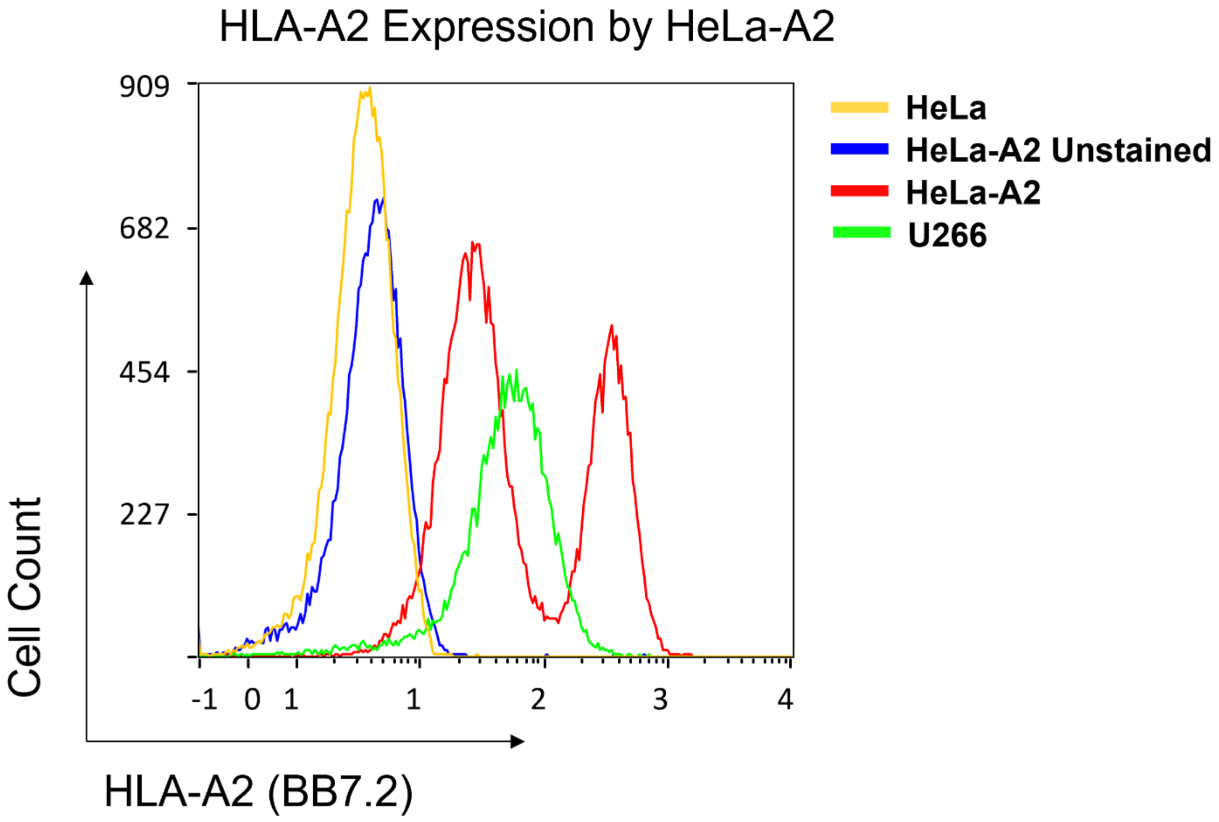

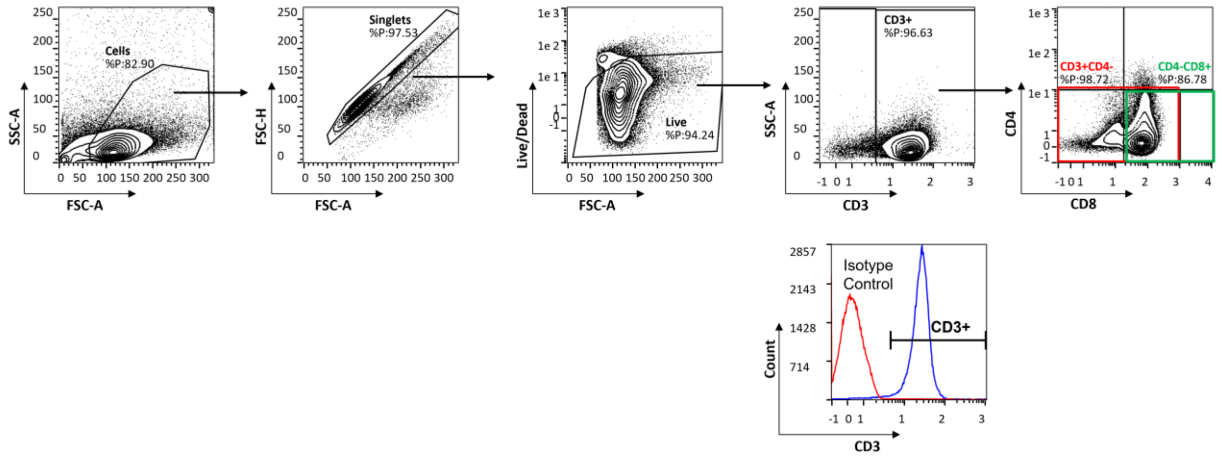
Cells
%P:82.90
SSC-A
FSC-A
Singlets
%P:97.53
FSC-H
FSC-A
Live
%P:94.24
Live/Dead
FSC-A
CD3+
%P:96.63
SSC-A
CD3
CD3+CD4-
%P:98.72
CD4-CD8+
%P:86.78
CD4
CD8
Isotype
Control
CD3+
Count
CD3

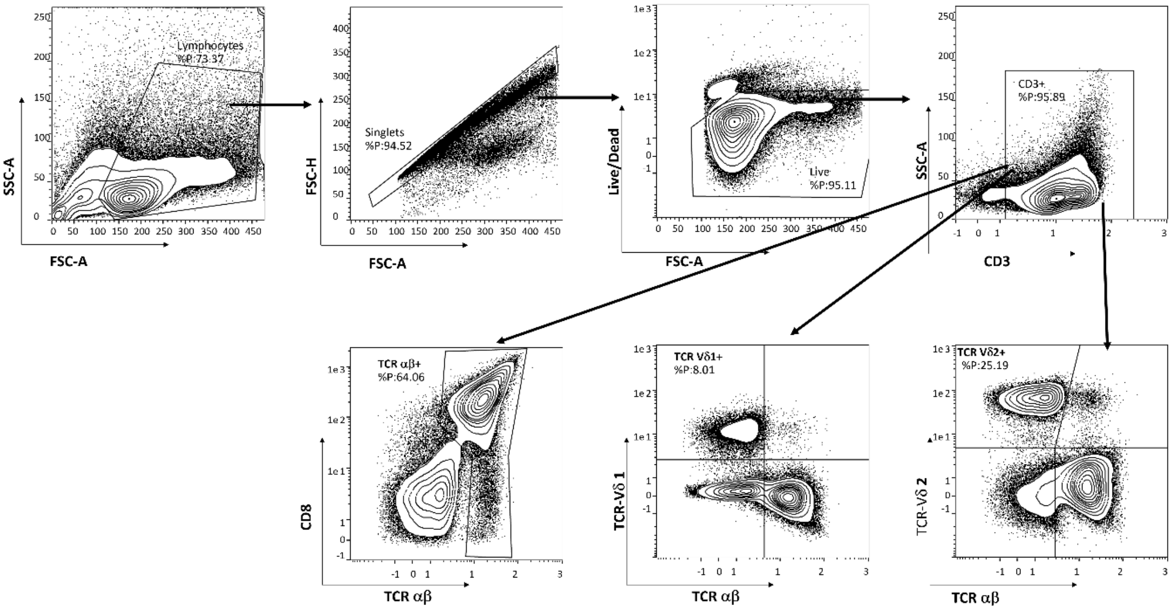
Lymphocytes
%P:73.37
SSC-A
FSC-A
Singlets
%P:94.52
FSC-H
FSC-A
Live
%P:95.11
Live/Dead
FSC-A
CD3+
%P:95.89
SSC-A
CD3
TCR αβ+
%P:64.06
CD8
TCR αβ
TCR Vδ1+
%P:8.01
TCR-Vδ 1
TCR αβ
TCR Vδ2+
%P:25.19
TCR-Vδ 2
TCR αβ

AIM platform Therapies: AIM nanoparticles for either adoptive cell therapies or as an “off-the-shelf” injectable therapeutic

A different nanoparticle core, but the same T cell engagement, signaling and multi-antigen targeting

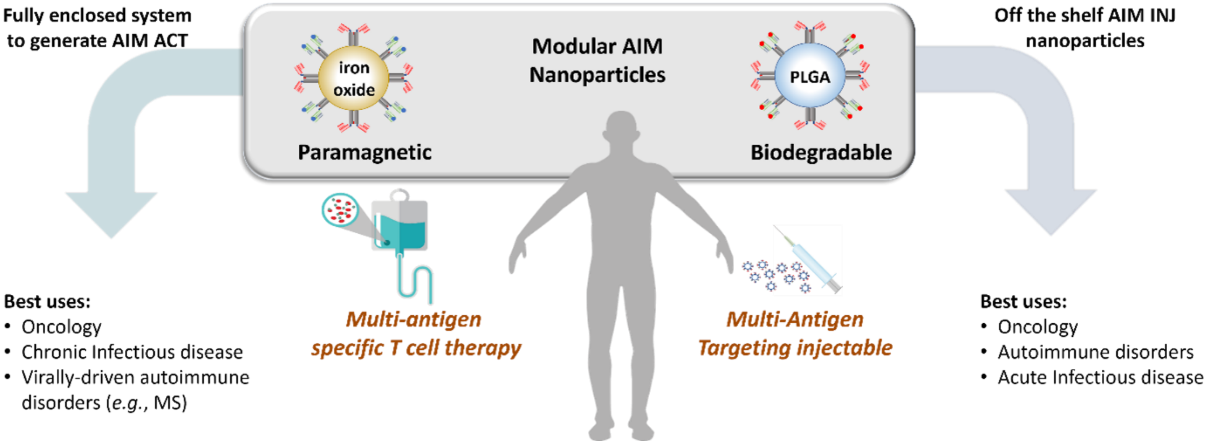

Supplementary Figure Legends

Supplementary Figure 1. HeLa-A2 cells express HLA-A2. Cells were stained for HLA-A2 expression using anti-human HLA-A2 monoclonal antibody clone BB7.2. Expression by the parental HeLa cell line (yellow line), the HeLa-A2 cell line (red line), and U266 (green line, a human HLA-A*02:01 cell line) cell line. HeLa-A2 left unstained (blue line).

Supplementary Figure 2. Identity Panel gating strategy for T cell subset frequencies. Gating of total T cells ($CD3^+$), $CD8^+$ T cells ($CD3^+$/$CD4^-CD8^+$), and $CD4^-$ T cells ($CD3^+$/$CD4^-$).

Supplementary Figure 3. TCR Panel gating strategy for Vδ subset frequencies. Gating of Vδ1 T cells (Vδ1+αβ-) and Vδ2 T cells (Vδ2+αβ-).

Supplementary Figure 4. AIM platform therapies: AIM nanoparticles for either adoptive cell therapies (ACT) or as an off-the-shelf injectable therapeutic (INJ). The AIM nanoparticle can be used as a paramagnetic AIM nanoparticle *ex vivo* to enrich and expand multi-antigen targeted T cells for the AIM ACT product, or as an injectable biodegradable AIM nanoparticles *in vivo* for activating and expanding multi-antigen targeting T cells.
